# Correlation between KRAS and NRAS mutational status and clinicopathological features in 414 cases of metastatic colorectal cancer in Morocco: the largest North African case series

**DOI:** 10.1186/s12876-023-02694-7

**Published:** 2023-06-05

**Authors:** Youssef Mahdi, Mouna Khmou, Amine Souadka, Hajar El Agouri, Soumaya Ech-charif, Chaimaa Mounjid, Basma El Khannoussi

**Affiliations:** 1 Pathology department, National Institute of Oncology, Rabat, Morocco; 2 Faculty of Medicine and Pharmacy, Mohammed V University in Rabat, Rabat, Morocco; 3 Surgical Oncology department, National Institute of Oncology, Rabat, Morocco; 4 Laboratory of Biology of Human Pathologies (BioPath), Faculty of sciences, Mohammed V University in Rabat, Rabat, Morocco

**Keywords:** KRAS, NRAS, Colorectal neoplasms, Metastases, North Africa.

## Abstract

**Background:**

Advances in molecular biology have improved understanding of the molecular features of carcinogenesis and progression of colorectal cancer. It is clear that the efficacy of anti-EGFR depends upon the RAS mutational status, since any mutation in RAS is associated with resistance to anti-EGFR therapy. The aim of this study is to report the largest North African description of KRAS and NRAS status in metastatic colorectal cancer and to describe the association of these mutations with clinicopathological characteristics.

**Methods:**

This is a prospective study of all consecutive unselected metastatic colorectal cancer samples, collected from the Laboratory of Pathology at the National Institute of Oncology of Rabat, Morocco, from January 1st 2020 to December 31st 2021. The molecular analysis was performed on the Idylla™ platform (fully automated real-time polymerase chain reaction-based assay) for KRAS and NRAS mutations in exons 2, 3 and 4. These mutations were correlated to gender, primary tumor site, histological type and degree of differentiation of tumor using adequate statistical methods.

**Results:**

Four hundred fourteen colorectal tumors were screened for KRAS and NRAS mutations. These mutations occurred in 51.7% of tumors for KRAS (mainly in exon 12) and in 3% of tumors for NRAS. There was a significant correlation between NRAS mutation and age of colorectal patients in this study. The low rate of invalid RAS tests (1.7% for KRAS and 3.1% for NRAS) was certainly obtained due to the strict respect of pre-analytical factors such as cold ischemia time and formalin fixation.

**Conclusion:**

We report the largest North African analysis of NRAS and KRAS status in colorectal metastatic patients. This study showed the ability in low middle income countries to perform a high rate of valid tests and the unusual trend towards older patients for NRAS mutations.

## Background

Colorectal cancer (CRC) is the second most common cause of cancer deaths [1]. Approximately 20% of patients with CRC present with metastases at the time of diagnosis [1]. Metastatic and recurrent diseases are associated with poor survival [2]. Advances in molecular biology have improved understanding of the molecular features of carcinogenesis and progression of CRC. Thus, the identification of prognostic and predictive biomarkers made it possible to refine prognosis in order to prescribe targeted therapies as part of a personalized medicine. In this context, it has been demonstrated that Epidermal growth factor receptor (EGFR) signaling is an important intracellular signaling pathway in CRC development and progression. RAS (for rat sarcoma virus) proteins are downstream signaling molecules of EGFR [3, 4]. There are three isoforms of RAS: KRAS, NRAS and HRAS [3, 4]. The efficacy of anti-EGFR depends upon the RAS mutational status. Mutations in RAS are associated with resistance to anti-EGFR therapy [3, 5–8]. HRAS is rarely mutated in CRC [3]. Treatment guidelines now recommend that all metastatic CRC (mCRC) patients should be tested for KRAS and NRAS if anti-EGFR therapy is available [9–11]. Only few studies were conducted in low middle income countries due to the high costs related to tumor mutation tests. Our first experience showed no statistically significant relationship between mCRC patient characteristics and KRAS/NRAS mutations. However due to low samples no conclusion has been made. The aim of this study was to report KRAS and NRAS mutation status frequencies in Moroccan patients with mCRC and investigate the associations of these mutations with clinicopathological characteristics in a larger study.

## Methods

This study represents the second report of our pilot study in Morocco using the Idylla™ platform: a fully automated, real-time PCR based molecular diagnosis system. Short-term laboratory-based training of technicians for one day only was necessary.

### Patients and tumor samples

This is a prospective study where data of consecutive unselected CRC samples were collected from the Laboratory of Pathology at the National Institute of Oncology of Rabat, Morocco, from January 1st 2020 to December 31st 2021. Only patients with confirmed histopathological metastatic CRC were included. Formalin-fixed, paraffin-embedded (FFPE) tumor sections obtained from biopsies or surgical resection were stained with hematoxylin and eosin (H&E). A trained pathologist (YM, MK, SE or BE) confirmed the diagnosis and identified the histological type and differentiation degree of the tumor. He also verified that at least 10% of viable tumor cells were present in every H&E stained slide. If so, each test included one to five 5–10 μm sections of FFPE tissue (5 to 25 μm thickness were needed according to the recommendations for pre-analytical preparation of FFPE samples prior to Idylla™ KRAS and NRAS mutation testing). If not, macrodissection was performed for tumor enrichment. It consists of FFPE tissue manual dissection using scalpel or pipette tip to remove microscopically tumor areas marked by a pathologist on the slide.

### KRAS and NRAS mutations analysis

FFPE sections were inserted directly into the Idylla™ platform. Extraction and PCR analysis were performed on the Idylla™ system using cartridges with allele-specific primers that allow qualitative detection of mutations. KRAS and NRAS mutations in codons 12 and 13 of exon 2, 59 and 61 of exon 3 and 117 and 146 of exon 4 were analyzed.

### Statistical analysis

The characteristics such as gender, primary tumor site, histological type and degree of tumor differentiation were reported in frequencies. All the continuous variables were reported as mean +/- Standard Deviation (SD) or median with quartiles.

The age was categorized into two categories choosing the cut-off of 60 years old according to the available literature.

The potential associations of KRAS and NRAS mutations with gender, primary tumor site, histological type and degree of differentiation of tumor were analyzed using chi-square (Χ²) test. Tests were considered statistically significant when p value was less than 0.05. All analyses were performed using SPSS 25.0.

## Results

### Clinicopathological characteristics of patients

**Patients**.

A total of 414 patients with metastatic CRC were enrolled in this study. 215 (52%) were men and 199 (48%) were women, with a mean age of 59 years ± 16.9 (range 23 to 90 years).

**Tumor specimens**.

Specimens were from primary tumor (n = 361, 87.2%) or metastases (n = 53, 12.8%) (mainly in liver, peritoneum and lung).

**Pathological characteristics**.

The rectum was the most frequent site of primary tumor (n = 145, 35%) followed by right-sided colon (n = 94, 22.7%) and sigmoid colon (n = 84, 20.3%). Site was not specified for 3 cases.

Adenocarcinoma not otherwise specified (NOS) was the most frequent histological type (90%). Other subtypes were mucinous adenocarcinoma, poorly cohesive carcinoma and neuroendocrine small cell carcinoma.

Tumors were mostly well or moderately differentiated.

The clinicopathological characteristics of the patients are summarized in Table 1.

**Table 1:** Clinicopathological features of patients

	N	%	
Age, years			
≤ 60	198	47.8	
> 60	216	52.8	
Gender			
Female	199	48	
Male	215	52	
Tumor tissue			
Primary	361	87.2	
Metastasis	53	12.8	
Liver	29	7	
Peritoneum	11	2.7	
Lung	8	1.9	
Bladder	3	0.7	
Ovary	2	0.5	
Primary tumor site			
Left-sided colon	300	72.5	
Right-sided colon	111	26.8	
Not specified	3	0.7	
Histological type			
Adenocarcinoma, NOS	373	90	
Mucinous adenocarcinoma	34	8.2	
Poorly cohesive carcinoma	4	1	
Neuroendocrine small cell carcinoma	3	0.7	
Differentiation			
Well differentiated	155	37.4	
Moderately differentiated	224	54.1	
Poorly differentiated	35	8.5	
RAS Status			
Mutation	226	54.6	

### Mutational status

Analysis was successful in 407 cases (98.3%) for KRAS. Invalid RAS tests were 1.7% for KRAS and 3.1% for NRAS.

KRAS mutation was detected in 51.7% (n = 214/414) of tumors, mainly in codon 12 (78.5%, n = 168). More than 50% of mutations were c.35G > T or c.35G > A (Table 2). These results are concordant to those previously described in the North African population.

**Table 2:** KRAS mutation status and frequency of mutation types

	N	%
**Status**		
Mutation	214	51.7
Wild-type	193	46.6
Unknow (invalid test)	7	1.7
**Mutation**		
Codon 12 c.35G > T (p.Gly12Val) c.35G > A (p.Gly12Asp) c.34G > T (p.Gly12Cys) c.34G > C (p.Gly12Arg) c.34G > A (p.Gly12Ser) c.35G > C (p.Gly12Ala)	168 61 54 23 11 10 9	78.5 28.5 25.2 10.7 5.1 4.7 4.2
Codon 13 c.38G > A (p.Gly13Asp)	25	11.7
Codon 146 c.436G > C/c.436G > A/c.437 C > T (p.Ala146Pro/p.Ala146Thr/p.Ala146Val)	16	7.5
Codon 59 c.175G > A / c.176 C > A / c.176 C > G (p.Ala59Thr/p.Ala59Val/ p.Ala59Gly)	1	0.5
Codon 61 c.182 A > G (p.Gln61Arg)	4	1.9

For NRAS, analysis was successful in 401 cases (96.9%). 94% (389/414) of tumors had wild-type NRAS. NRAS mutation was detected only in 3% (12/414) of cases: codon 61 (8 cases), codon 12 (3 cases) and codon 13 (1 case) (Table 3).

**Table 3:** NRAS mutation status and frequency of mutation types

	N	%
**Status**		
Mutation	12	2.9
Wild-type	389	94
Unknow (invalid test)	13	3.1
**Mutation**		
Codon 12 c.35G > C (p.Gly12Ala) c.35G > T (p.Gly12Val) c.35G > A (p.Gly12Asp)	3 1 1 1	25 8.3 8.3 8.3
Codon 13 c.38G > A (p.Gly13Asp)	1	8.3
Codon 61 c.182 A > G (p.Gln61Arg) c.181 C > A (p.Gln61Lys) c.182 A > T (p.Gln61Leu) c.183 A > T (p.Gln61His)	8 3 2 2 1	66.7 25 16.7 16.7 8.3

### Associations between RAS and clinicopathological features

A summary of the main clinicopathological features of KRAS and NRAS mutants and wild-type CRC are shown in Tables 4 and 5.

**Table 4:** Association between KRAS status and clinicopathological features (n = 407/414)

	Mutation n (%)	Wild-type n (%)	p value
Age, years			0.28
≤ 60 (n = 195)	99 (50.8)	96 (49.2)	
> 60 (n = 212)	119 (56.1)	93 (43.9)	
Gender			0.45
Female (n = 195)	100 (51.3)	95 (48.7)	
Male (n = 212)	114 (53.8)	98 (46.2)	
Primary tumor site			0.08
Left-sided colon	150 (50.2)	149 (49.8)	
Right-sided colon	63 (60)	42 (40)	
Not specified	1 (0.33)	2 (0.67)	
Histological type			0.79
Adenocarcinoma, NOS (n = 366)	190 (51.9)	176 (48.1)	
Mucinous adenocarcinoma (n = 34)	20 (58.8)	14 (41.2)	
Poorly cohesive carcinoma (n = 4)	3 (75)	1 (25)	
Neuroendocrine small cell carcinoma (n = 3)	1 (33.3)	2 (66.7)	
Tumor differentiation			0.21
Well differentiated (n = 152)	83 (54.6)	69 (45.4)	
Moderately differentiated (n = 220)	119 (54.1)	101 (45.9)	
Poorly differentiated (n = 35)	12 (34.3)	23 (65.7)	

**Table 5:** Association between NRAS status and clinicopathological features (n = 401/414)

	Mutation n (%)	Wild-type n (%)	p value
Age, years			**0.005**
≤ 60 (n = 193)	1 (0.5)	192 (99.5)	
> 60 (n = 208)	11 (5.3)	197 (94.7)	
Gender			0.64
Female (n = 194)	5 (2.6)	189 (97.4)	
Male (n = 207)	7 (3.4)	200 (96.6)	
Primary tumor site			0.44
Left-sided colon	10 (3.4)	285 (96.6)	
Right-sided colon	2 (1.9)	103 (98.1)	
Not specified	0 (0)	1 (100)	
Histological type			0.49
Adenocarcinoma, NOS (n = 360)	12 (3.3)	348 (96.7)	
Mucinous adenocarcinoma (n = 34)	0	34 (100)	
Poorly cohesive carcinoma (n = 4)	0	4 (100)	
Neuroendocrine small cell carcinoma (n = 3)	0	3 (100)	
Tumor differentiation			0.79
Well differentiated (n = 150)	8 (5.3)	142 (94.7)	
Moderately differentiated (n = 217)	4 (1.8)	213 (98.2)	
Poorly differentiated (n = 34)	0	34 (100)	

KRAS and NRAS mutations were more frequent in older patients (56.1% and 5.3% respectively in > 60 years), than in the younger ones (50.8% and 0.5% respectively in ≤ 60 years). For NRAS, the mean age was significantly different between the two groups (p = 0.005). KRAS and NRAS mutations were slightly more common in men than in women (53.8% and 3.4 versus 51.3% and 2.6% respectively). Moreover, KRAS mutations were found more frequently in right-sided (60%) than in left-sided (50.2%) colon cancer, but this did not reach statistical significance (p = 0.08). In contrast, NRAS mutation was more frequent in left-sided colon cancer (3.4% versus 1.9%), but also this did not reach statistical significance (p = 0.44).

KRAS and NRAS mutations were found respectively in 52.9% (190/359) and 3.4% (12/353) of primary tumors and 50% (24/48) and 0% for metastases. These results did not reach statistical significance (p = 0.7 for KRAS and p = 0.19 for NRAS).

For histological type, frequency of KRAS mutation was higher in poorly cohesive carcinoma (75%, n = 3/4), than in other histological types (58.8% in mucinous adenocarcinoma, 51.9% in adenocarcinoma NOS). NRAS mutation was exclusively found in adenocarcinoma, NOS (3.3%, n = 12/360). All these findings did not reach statistical significance (p = 0.79 for KRAS and p = 0.49 for NRAS). KRAS mutation was found more in well and moderately (54.6% and 54.1% respectively) than in poorly differentiated carcinoma (34.3%). NRAS mutation was observed more in well (5.3%) than in moderately (1.8%) differentiated carcinoma. Interestingly, no NRAS mutation was found in poorly differentiated carcinoma (0/35). These findings also did not reach statistical significance (p = 0.21 for KRAS and p = 0.79 for NRAS).

## Discussion

This study showed RAS mutation in 54.6% of cases, a low rate of invalid cases despite being a pilot study, significant correlation between NRAS mutation and age and no correlations between RAS mutations and other clinicopathological characteristics.

Previous studies have demonstrated highly variable frequencies of RAS mutations in North Africa ranging from 16 to 75.2% [12–25] (80% for one study but with 20 patients only) [26].

RAS mutation is an early event in colorectal carcinogenesis. In this study, most of tissues (87.20%) were from primary tumor. This high frequency of primary tumor on specimens for RAS study is probably due to habitudes of our institute to not systematically re-biopsy for metastases or rapid progression of disease. Although a high concordance exists between primary CRC and liver and lung metastases [27, 28], metastatic or recurrent CRC tissues are the preferred specimens for RAS (and other biomarkers) testing [29]. Indeed, acquired KRAS mutations during progression of CRC metastases can occur [30]. In absence of metastatic and recurrent CRC tissues, primary tumor tissue is an acceptable alternative [29, 30].

We obtained 20 cases with invalid RAS results: 7 (1.7%) for KRAS and 13 (3.1%) for NRAS. We think that this low rate of invalid cases was achieved through respect to pre-analytical factors: cold ischemia time and formalin fixation. All invalid cases corresponded to prepared outside of the laboratory paraffin blocks. We think that a probable non-optimal pre-analytical phase may have caused irreversible DNA and RNA damage. This made their amplification impossible.

For correlation of primary tumor localization and RAS mutation, literature also was controversial. KRAS was significantly more frequent in left-sided CRC in three studies [20–22]. In our study, no correlation between primary tumor site and RAS mutation was observed. Data for association between RAS status and histological type and tumor differentiation were also inconclusive. A summary of frequency and association between RAS status and clinicopathological features in North Africa are shown in Table 6.

**Table 6:** Frequency and association between RAS status and clinicopathological features in North Africa

	RAS mutation %	Age	Gender	Primary tumor site	Histological type	Tumor differentiation
El-Serafi et al. [12] (n = 90)	KRAS = 41.1% (cod 12 + 13)	NS	N/A	N/A	N/A	N/A
Bennani et al. [13] (n = 62)	KRAS = 29% (cod 12 + 13)	NS	NS	NS	N/A	N/A
Karim et al. [14] (n = 48)	KRAS = 45.83% (cod 12 + 13)	N/A	N/A	N/A	N/A	N/A
Sammoud et al. [15] (n = 52)	KRAS = 23.07% (cod 12 + 13)	NS	**Female gender** (P = 0.017)	NS	NS	N/A
Kamal et al. [16] (n = 80)	KRAS = 28.75%	NS	NS	N/A	N/A	N/A
Aissi et al. [17] (n = 51)	KRAS = 31.5% (cod 12 + 13)	NS	NS	NS	N/A	N/A
Marchoudi et al. [18] (n = 92)	KRAS = 23.91% (cod 12 + 13)	NS	N/A	NS	N/A	NS
Ines et al. [19] (n = 167)	KRAS = 31.13% (cod 12 + 13)	NS	**Female gender** (P = 0.008)	NS (colon vs. rectum)	NS	N/A
Jouini et al. [20] (n = 131)	KRAS = 68.22% NRAS = 6.97% (RAS = 75.2%)	NS	NS	NS	NS	**For mutation class (KRAS exon 2 vs. outside KRAS exon 2**, p = 0.012**)**
Ounissi et al. [21] (n = 96)	KRAS = 41.79% (cod 12 + 13) NRAS = 7.3% (exons 2,3,4)	**Older** patients* (p = 0.029)	NS	Left colon* (p = 0.037)	NS	Greater differentiation* (p = 0.044)
El Agy et al. [22] (n = 210)	KRAS = 36.7% NRAS = 2.9% (RAS = 39.5%)	NS	**Female gender** (p = 0.003)	**Left colon** (p = 0.009)	**Classical ADC (p = 0.01)**	**Moderately differentiated** (p = 0.04)
Abudabous et al. [23] (n = 34)	KRAS = 38.2% HRAS = 0% (cod 12 + 13)	NS	NS	**Left colon** (p = 0.027)	N/A	NS
El Asri et al. [24] (n = 151)	KRAS = 34.4% (cod 12 + 13)	NS	NS	NS (colon vs. rectum)	N/A	N/A
Salah El-Din Youssef et al. [25] (n = 45)	KRAS = 16%	N/A	N/A	N/A	N/A	N/A
Abd El Kader et al. [26] (n = 20)	KRAS = 80% (cod 12 + 13)	N/A	N/A	N/A	N/A	N/A

N/A : Not available.

NS : Association not statistically significant.

Cod : codons.

* : for KRAS exon 2.

ADC : Adenocarcinoma.

Our preliminary results did not show a statistically significant relationship between clinicopathological features and KRAS and NRAS mutations (n = 186) [31]. In the present study, there was a trend towards older patients (> 60 years) for NRAS mutations. Indeed, only one patient with NRAS mutation has ≤ 60 years (n = 1/12).

Worldwide, distribution of RAS mutations is uneven. The KRAS mutation rate is 44.7% in Western Europe, 35.8% in Eastern Europe [32], 41% in Indonesia [33] and 36.1% in China [34] (for Asia) and 19.5% in the Middle East [32]. In many studies, KRAS mutation incidence was higher in right-sided than in left-sided colon tumors [34–36] and higher in women [34, 36, 37] but not for NRAS mutation [34, 35]. Other studies did not find differences in location of primary tumor [37, 38] and patients’ gender [38] for KRAS mutation prevalence. Interestingly, KRAS was mutated significantly more often in the primary tumors of patients with lung metastases in one study [27].

This study has several limitations:

1) The small number of samples compared to the published literature from Western countries. However, it represents the largest North African case series.

2) The use of the Idylla™ platform. Indeed, Next Generation Sequencing (NGS) is the current diagnostic gold-standard for RAS mutational analysis in CRC. Several studies have demonstrated that Idylla™ testing is highly accurate with excellent concordance with NGS [39–41]. In addition, Idylla™ testing is less than 2 min hands-on time and rapid (2 h) compared with NGS. Costs are lowest for Idylla™ making molecular testing affordable (no molecular pathology platform is needed). This can be very useful for low- and middle-income countries, especially for a small number of cases to test. Our laboratory is in the process of setting up a molecular pathology platform. Our perspective is to retest all 414 specimens with NGS to determine accuracy of our technique.

3) The main objective of the study did not include the analysis of survival and recurrence but this is consistent with this first pilot study of feasibility and assessment of RAS mutations. With more hindsight we will be able to report survival and recurrence of this case series.

## Conclusion

We report the largest North African analysis of NRAS and KRAS status in colorectal metastatic patients. This study showed the ability in low middle income countries to perform a high rate of valid tests and the unusual trend towards older patients for NRAS mutations.

## Data Availability

The datasets used and/or analyzed during the current study available from the corresponding author on reasonable request.

## List of abbreviations

CRC: Colorectal cancer
EGFR: Epidermal growth factor receptor
RAS: Rat sarcoma virus
PCR: Polymerase chain reaction
FFPE: Formalin-fixed, paraffin-embedded
NOS: Not otherwise specified
NGS: Next Generation Sequencing.
